# Clinically diagnosed tetanus in a 58-year-old female with breast fungating mass

**DOI:** 10.1186/s41182-025-00878-3

**Published:** 2025-12-19

**Authors:** Maria Fe R. Cruz, Chris Smith, Kensuke Takahashi, Su Myat Han, Jeffrey A. Verona

**Affiliations:** 1 Adult Infectious Disease and Tropical Medicine Department, San Lazaro Hospital, Manila, Philippines; 2 School of Tropical Medicine and Global Health, Nagasaki University, Nagasaki, Japan; 3 Department of Clinical Research, London School of Hygiene and Tropical Medicine, London, UK; 4 San Lazaro Hospital-Nagasaki University Collaborative Research Office, San Lazaro Hospital, Manila, Philippines; 5 Acute and Critical Care Unit, Nagasaki University Hospital, Nagasaki, Japan; 6 Department of Infectious Disease Epidemiology and Dynamics, Institute of Tropical Medicine, Nagasaki University, Nagasaki, Japan

**Keywords:** Tetanus, Breast mass, Necrotic wound, Trismus

## Abstract

**Background:**

Tetanus remains a rare but potentially fatal disease, typically associated with traumatic wounds. However, necrotic malignancies such as fungating breast tumors may also serve as an entry point for *Clostridium tetani* infection.

**Case presentation:**

We report the case of a 58-year-old female with a 3-year history of a fungating left breast mass who presented with trismus. A diagnosis of tetanus was made clinically. The patient received treatment with anti-tetanus globulin, metronidazole, and she was placed in a dark room with sound insulation and shielding. The surgical team was consulted for wound management. However, in accordance with the patient’s refusal, surgical debridement was not performed. Instead, local wound cleansing and supportive management were initiated.

**Conclusions:**

Tetanus should be considered in patients with necrotic tumors presenting with trismus, especially in low-resource settings where immunization histories are uncertain. Early intervention is crucial to reduce morbidity and prevent complications.

## Background

Tetanus is a neurological condition caused by the neurotoxin tetanospasmin, produced by *Clostridium tetani* [1, 2]. Clinically, tetanus is classified into four forms: generalized, localized, cephalic, and neonatal. Generalized tetanus is the most common and is characterized by trismus, generalized rigidity, and reflex spasms. Localized tetanus presents with muscle rigidity confined to the region of injury, while cephalic tetanus, typically following head or neck wounds, involves cranial nerve palsies. Neonatal tetanus occurs in newborns due to umbilical stump contamination. Pathophysiologically, tetanospasmin acts at the inhibitory synapses of the central nervous system. After entering peripheral nerves at the wound site, the toxin undergoes retrograde axonal transport to the spinal cord and brainstem, where it cleaves vesicle-associated membrane protein (VAMP, also known as synaptobrevin), a key component of the SNARE complex involved in neurotransmitter release. This inhibition prevents the exocytosis of inhibitory neurotransmitters γ-aminobutyric acid (GABA) and glycine, leading to unchecked excitation of motor and autonomic neurons and the characteristic muscle rigidity and spasms of tetanus [1, 2]. Though most cases are associated with trauma, the disease may also result from chronic, necrotic wounds, including malignant tumors [3–8]. Ulcerated or necrotic breast cancers are potential sources of infection, particularly when secondary bacterial colonization occurs under anaerobic conditions.

Despite global immunization efforts, tetanus remains a significant concern in many low- and middle-income countries, especially where access to immunization or wound care is limited [9]. In the Philippines, tetanus continues to be reported among unvaccinated individuals, those with chronic wounds, or in cases like this, where untreated malignancy provides an ideal environment for the bacteria.

### Case presentation

A 58-year-old female presented to an infectious disease referral hospital with a 2-day history of inability to open her mouth. She denied any recent trauma, fever, or systemic symptoms. She had a 3-year history of a progressively enlarging, bleeding, and foul-smelling left breast mass for which she had not sought medical attention. She had no known history of diabetes mellitus, hypertension, tuberculosis, HIV infection, or other chronic immunosuppressive conditions. There was no history of steroid use, chemotherapy, or radiotherapy prior to admission. She was referred to San Lazaro Hospital after a dental consult ruled out temporomandibular joint dysfunction.

**Fig. 1:**
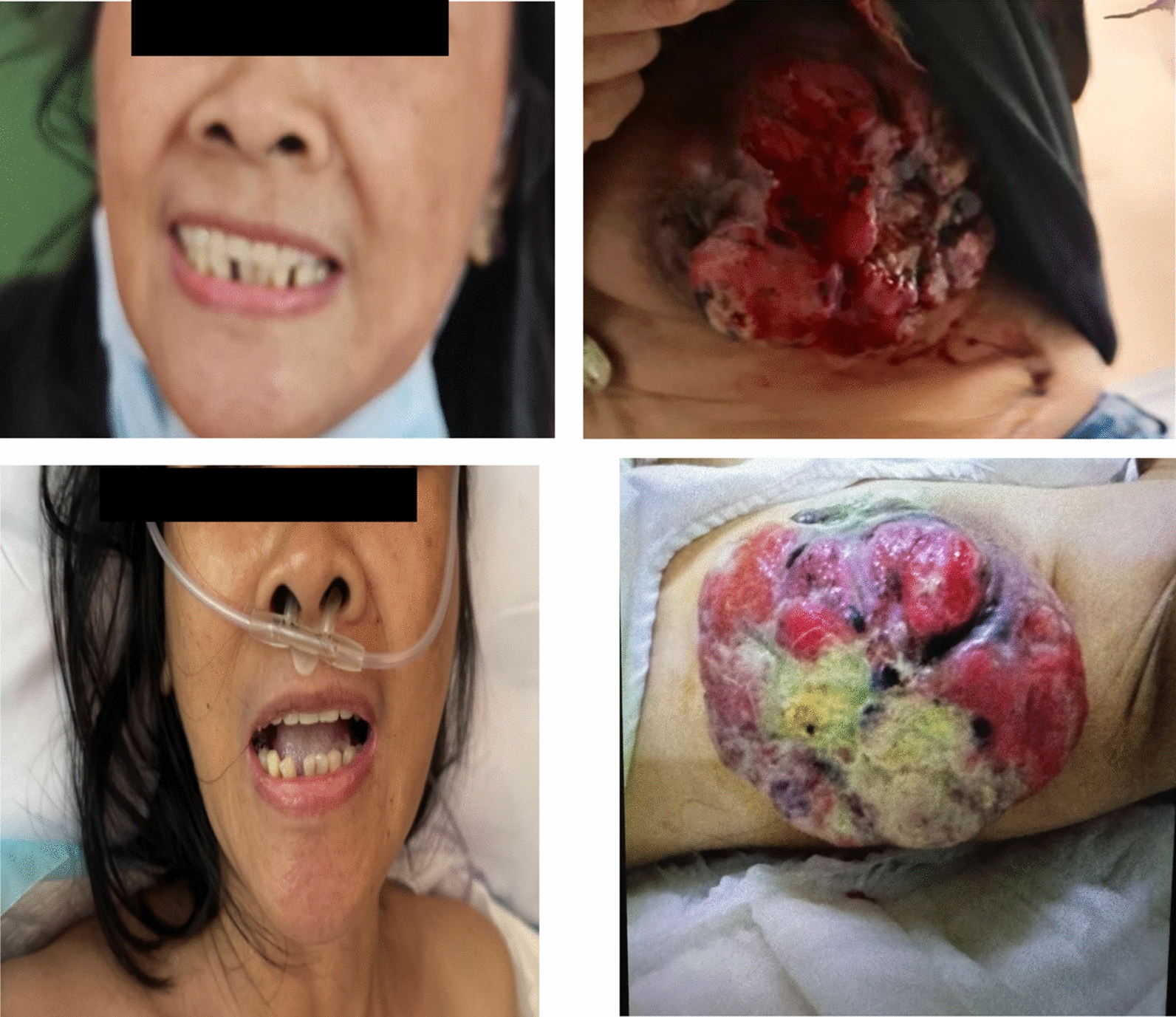
Figure showing trismus and breast mass upon admission (upper panel) and after 3 days of treatment (lower panel)

During admission, she was awake and alert (GCS 15) but in moderate distress due to trismus. Her vital signs were stable (BP 120/80 mmHg, HR 126 bpm, RR 22, T 36.4 °C, SpO₂ 98% on room air). Physical examination revealed a locked jaw and an erythematous, necrotic, bleeding breast mass (Fig. 1). There was no lymphadenopathy or focal neurological deficit. As there was no history of reentry trauma, the breast mass ulcer was considered the most likely source of *C. tetani* entry. A clinical diagnosis of tetanus was made.

Blood tests revealed a mild anemia, while other parameters, including inflammatory markers such as white blood cell count, remained within normal limits, suggesting no systemic infection but possible chronic inflammation related to the skin ulcer. The laboratory blood workups are summarized in Table 1.

**Table 1:** Laboratory findings

Parameter	Result	Reference range
WBC (× 10⁹/L)	9.1	4.0–10.0
Hemoglobin (g/L)	98	130–180
Platelets (× 10⁹/L)	189	150–400
Creatinine (µmol/L)	86	71–133
Sodium (mmol/L)	141	137–145
Potassium (mmol/L)	3.8	3.5–5.1
ALT (U/L)	23	< 50
AST (U/L)	27	17–59

On Day 1 of admission, the patient was given an intravenous injection of human tetanus immunoglobulin (20,000 IU), an intramuscular injection of tetanus toxoid (0.5 mL), metronidazole (500 mg every 8 h) and ampicillin–sulbactam (3G IV every 6 h), and benzodiazepine (10 mg every 8 h), and was placed in a dark, sound- and light-shielded room for tetanus management.

Over the next few days, the patient developed provoked spasms and abdominal muscle rigidity. However, the intensity of the spasms gradually decreased over time. By Day 3, her trismus improved, and she tolerated oral intake for the first time since symptom onset. According to the Ablett classification [10], the case was categorized as Grade II (moderate).

Wound care involved cleaning with normal saline followed by the application of a moist saline gauze dressing (often termed a wet-to-moist approach) three times daily. This involved applying saline-moistened gauze to the wound and removing it while it was still moist, with the frequent changing schedule (three times daily) specifically intended to prevent the gauze from drying out completely and adhering to healthy granulation tissue. This approach facilitated moist mechanical debridement while mitigating the trauma and re-injury risk associated with truly dry gauze removal. The surgical service instructed the patient on this wound care procedure. The patient gradually improved and was discharged on Day 10 following completion of the antibiotic course. Upon discharge, she was advised to follow up at the outpatient department (OPD) for the second dose of tetanus toxoid. She was also referred to Jose R. Reyes Memorial Medical Center for further management of her breast mass.

In establishing the diagnosis, other causes of trismus and muscle rigidity were considered. Neuroleptic malignant syndrome (NMS) was excluded because the patient had no history of antipsychotic or dopamine-antagonist drug use, hyperthermia, or altered sensorium. Stiff-person syndrome, an autoimmune disorder associated with anti-GAD antibodies, was considered unlikely given the acute onset, presence of wound-related exposure, and rapid progression of spasms. Strychnine poisoning was excluded based on the absence of toxin exposure history and the subacute rather than fulminant course. Hypocalcemia and drug-induced dystonias, including tardive dyskinesia, were ruled out by normal electrolyte results and lack of offending medication history. Collectively, these exclusions, combined with the characteristic trismus, risus sardonicus, and provoked spasms, supported the clinical diagnosis of tetanus.

## Discussion and conclusions

Tetanus remains a threat in settings with inadequate vaccine coverage, limited wound care, and delayed access to medical services. This case highlights a rare but increasingly reported presentation of tetanus associated with neglected, fungating breast malignancies. Necrotic tumor tissue provides an ideal anaerobic environment for *C. tetani* to thrive and produce toxin [4–8, 11].

Although surgical debridement is widely regarded as a cornerstone of tetanus management to eliminate the anaerobic environment and reduce the bacterial load, it was not performed in this case due to the patient’s explicit refusal of a surgical procedure. This decision, confirmed by the hospital’s Infectious Disease team, highlights a significant limitation of this case report. However, the patient responded favorably to a combination of tetanus immunoglobulin, antibiotics, supportive care, and local wound management, suggesting that in select cases without severe autonomic involvement, non-surgical management may suffice when absolute contraindications, such as patient refusal, are present.

Although neuroimaging (CT or MRI) can be useful to exclude other intracranial causes of rigidity or trismus, such as brainstem lesions, dystonic syndromes, or structural abnormalities, it was not performed in this case. In many low- and middle-income country (LMIC) settings, access to advanced imaging is limited and is generally reserved for patients with atypical or focal neurological findings. Similarly, laboratory tests for metabolic causes such as hypocalcemia, hypophosphatemia, or vitamin D deficiency were not obtained, as these assays are not routinely available at San Lazaro Hospital and are typically requested only when clinically indicated. The absence of focal deficits, altered consciousness, paresthesia, carpopedal spasm, or seizures, along with normal electrolytes, made both intracranial and metabolic etiologies unlikely. Thus, a clinical diagnosis of tetanus was considered sufficient, consistent with accepted diagnostic practice in resource-limited settings where diagnosis remains primarily clinical.

Standard wound management for suspected or confirmed tetanus exposure involves both passive and active immunization. According to World Health Organization and Philippine Department of Health guidelines, human tetanus immunoglobulin (TIG) should be administered at a dose of 3000–6000 IU intramuscularly, ideally at a site separate from the vaccine injection, to provide immediate passive immunity by neutralizing circulating toxin [12, 13]. Tetanus toxoid (0.5 mL intramuscularly) should be given concurrently to initiate active immunization and stimulate long-term antibody production. In individuals with uncertain or incomplete vaccination history and contaminated or “dirty” wounds, a three-dose primary series is recommended—an initial dose at presentation, followed by booster doses at 1 month and 6–12 months after the first dose. This combined approach ensures both immediate and durable protection and is the cornerstone of tetanus prevention in wound management [12, 13].

In the present case, immunization history was unknown, raising concern about whether she possessed any pre-existing immunity. Given the potential for *C. tetani* spores to remain latent in necrotic tissue, the risk of recurrence must be carefully considered. As reported in previous cases, a single dose of toxoid may not provide sufficient protection, particularly in patients with cancer-related immunosuppression [14, 15]. In accordance with Philippines department of health (DoH) guideline, the patient was advised to complete a three-dose booster series at 1 month, 6 months, and 1 year post-discharge to ensure adequate long-term protection [12, 13].

This case underscores several key messages: tetanus should be considered in patients presenting with trismus and chronic necrotic wounds, even in the absence of trauma; diagnosis remains clinical in resource-limited settings; and when surgical debridement is not feasible, a combination of early immunoglobulin, antimicrobial therapy, wound care, and active immunization may still achieve remission. Increased awareness is needed among clinicians regarding atypical presentations of tetanus, particularly in oncology patients with ulcerative tumors.

## Data Availability

All data generated or analyzed during this study are included in this published article.

## Abbreviations

TIG: Tetanus immunoglobulin
GCS: Glasgow Coma Scale
